# The Impact of Primary Tumor and Locoregional Metastatic Lymph Node SUV_max_ on Predicting Survival in Patients with Rectal Cancer

**DOI:** 10.4274/mirt.galenos.2020.40316

**Published:** 2020-04-29

**Authors:** Göksel Alçın, Yasemin Şanlı, Gülçin Yeğen, Esra Kaytan Sağlam, Tevfik Fikret Çermik

**Affiliations:** 1 University of Health and Sciences, İstanbul Training and Research Hospital, Clinic of Nuclear Medicine, İstanbul, Turkey; 2 İstanbul University, İstanbul Faculty of Medicine, Department of Nuclear Medicine, İstanbul, Turkey; 3 İstanbul University, İstanbul Faculty of Medicine, Department of Pathology, İstanbul, Turkey; 4 İstanbul University, İstanbul Faculty of Medicine, Department of Radiation Oncology, İstanbul, Turkey

**Keywords:** Rectal adenocarcinoma, 18F-FDG PET/CT, staging, SUVmax, prognosis

## Abstract

**Objectives:**

The aim of this study was to evaluate the impact of maximum standard uptake value (SUV_max_) of the primary tumor and locoregional metastatic lymph node in predicting survival in patients with the preoperative rectal adenocarcinoma.

**Methods:**

One hundred and fifteen patients [mean age ± standard deviation (SD): 58.7±11.4 years] with biopsy-proven rectal adenocarcinoma underwent ^18^F-fluorodeoxyglucose (FDG) positron emission tomography/computed tomography (PET/CT) imaging for the staging were included in this study. All patients were followed-up for a minimum of 12 months (mean ± SD: 29.7±13.5 months). Tumor-node-metastasis 2017 clinical staging, SUV_max_ of the primary rectal tumor and locoregional lymph nodes on the PET/CT studies were evaluated.

**Results:**

All patients had increased FDG activity of the primary tumor. The mean ± SD SUV_max_ of the primary tumor and locoregional metastatic lymph node were 21.0±9.1 and 4.6±2.8, respectively. Primary tumor SUV_max_ did not have an effect on predicting survival (p=0.525) however locoregional metastatic lymph node SUV_max_ had an effect (p<0.05) on predicting survival. Clinical stage of the disease was a factor predicting survival (p<0.001).

**Conclusion:**

^18^F-FDG PET/CT is an effective imaging modality for detecting primary tumors and metastases in rectal adenocarcinoma and clinical stage assessment with PET/CT had an effect on predicting survival. Furthermore, in our study locoregional lymph node SUV_maks_ was defined as a factor in predicting survival.

## Abstract (tr)

**Amaç:**

Çalışmamızın amacı preoperatif rektum adenokanserli olan hastalarda sağkalımı öngörmede primer tümör ve lokorejyonel metastatik lenf nodu maksimum standart alım değeri (SUV_maks_) değerinin etkinliğini değerlendirmektir.

**Yöntem:**

Bu çalışmaya, biyopsiyle kanıtlanmış rektal adenokarsinoma ile evreleme amaçlı ^18^F-florodeoksiglikoz (FDG) pozitron emisyon tomografi/bilgisayarlı tomografi (PET/BT) görüntüleme yapılan 115 hasta [ortalama yaş ± standart sapma (SS): 58,7±11,4 yıl] dahil edildi. Tüm hastalar en az 12 ay (ortalama ± SS: 29,7±13,5 ay) takip edildi. Tümör-nodül-metastaz 2017 klinik evreleme, primer rektal tümör SUV_maks_ ve lokorejyonel lenf nodu SUV_maks_değerlendirildi.

**Bulgular:**

Tüm hastalarda primer tümöre ait artmış FDG tutulumu saptandı. Primer tümör ve lokorejyonel metastatik lenf nodu ortalama SUV_maks_’ları sırasıyla 21,0±9,1 ve 4,6±2,8 idi. Primer tümör SUV_maks_’ın sağkalımı öngörmede bir etkisi saptanmamış olup (p=0,525) lokorejyonel metastatik lenf nodu SUV_maks_’ın sağkalımı öngörmede etkisi saptandı (p<0,05). Hastalığın klinik evresi sağkalımı öngören bir faktördü (p<0,001).

**Sonuç:**

^18^F-FDG PET/BT rektum kanserinde primer tümör ve metastazlarının saptanmasında etkili bir görüntüleme yöntemi olup lokorejyonel lenf nodu SUV_maks_ değerlerinin prognostik değerinin bulunduğu ve rektum kanserinin tedavi öncesi preoperatif evrelemede hastaların tedavi yönetimine önemli katkılar sağlayacağı düşünülmüştür.

## Introduction

Colorectal cancer is the third most common cancer in men and the second most common in women (1). Furthermore, it is one of the most important causes of cancer-related morbidity and mortality, globally (2). The primary treatment is surgical resection of the primary tumor and coupled with a (neo) adjuvant therapy increases the rates of survival of colorectal carcinoma (3). The importance of clinical staging with positron emission tomography/computed tomography (PET/CT) is established (4), the effect of the maximum standard uptake value (SUV_max_) acquired with ^18^F-fluorodeoxyglucose (FDG) PET/CT on survival in initial staging has been evaluated by several studies. Primary tumor SUV_max _is related to survival in some studies (5,6) however, some authors reported that primary tumor SUV_max_ was not associated with survival (7). On the other hand, the effect of metastatic locoregional lymph node FDG uptake level on survival has not been sufficiently studied. The aim of this study was to analyze the effect of the primary tumor and locoregional metastatic lymph node SUV_max_ and stage of disease detected with ^18^F-FDG PET/CT on the survival of patients with rectal adenocarcinoma.

## Materials and Methods

The study was approved by the Local Ethics Committee of İstanbul University İstanbul Medical Faculty of (2015/1867). The patients with histologically proven rectal adenocarcinoma who underwent FDG-PET/CT staging were assessed retrospectively. Clinical follow-up was performed until September 2015.

Localization of tumor was categorized as upper, mid rectal and distal rectal. Clinical staging was achieved using tumor-node-metastasis (TNM) staging provided by the American Joint Committee on Cancer Colon and Rectum Cancer Staging 8^th^ Edition after assessing pathology reports, CT and PET/CT images.

### PET/CT Protocol

### Patient Preparation

Patients with blood glucose levels lower than 200 mg/dL after at least 6 h of fasting were admitted for the procedure. Patients received an intravenous injection of 5.4 MBq/kg ^18^F-FDG and then rested for approximately 60 min before undergoing imaging. Patients were instructed to discontinue oral antidiabetic ‘metformin’ use 3 days before PET/CT imaging. Long-acting insulin treatment was allowed 12 hours before ^18^F-FDG injection. Patients were administered oral contrast media 12 hours before imaging. All of the patients were instructed to refrain any muscular activity to avoid muscle uptake during the distribution phase of injected ^18^F-FDG.

### PET/CT Imaging

PET/CT scan was performed using a Biograph 6 True Point HD LSO (Siemens Healthcare, Molecular Imaging, Knoxville, Tennessee, USA) integrated device. Initially, a CT scan (130-136 keV; 60-90 mAs) from vertex to upper thighs was performed in a single step which was used for attenuation correction of PET/CT images. The PET images were iteratively reconstructed with 5 mm thickness (TrueX option, subsets=21, iterations=3). A PET emission scan was acquired using whole-body mode following the CT scan. Six to 9 bed positions were used with an acquisition time of 3 minutes for each bed position. Additional imaging of the lower extremities was carried out in patients with multiple metastases. The PET data were reconstructed using 3D PET reconstruction with a system matrix derived from point source measurements.

### Image Analysis

All images were examined on an LCD monitor as attenuation-corrected and uncorrected multiplanar PET, CT and PET/CT fusion cross-sections (maximum intensity projection) using the eSOFT software. ^18^F-FDG-PET/CT studies were reviewed for abnormally increased tracer uptake foci by a nuclear medicine physician with minimum 5 years of experience. Each focal uptake identified in PET images was correlated in the corresponding CT sections and a PET/CT scan was considered to be positive if one or more areas of abnormal ^18^F-FDG uptake were noted with a corresponding abnormality in CT. Furthermore, focally increased ^18^F-FDG accumulations of lymph nodes higher than the background activity, a short axis of 6 mm or larger, round shaped and fatty hilum loss were considered as pathological lymph nodes. Pathological FDG uptake in the liver was assumed as metastasis. The quantification was made by calculating the SUV that was used as a relative measure of ^18^F-FDG uptake. The simple expression for SUV was the ratio of tracer activity concentration (C) in the region of interest and the decay-corrected amount of injected activity (kBq) per weight of the patient (gr): SUV=C (kBq/mL)/[injection activity (kBq)/patient’s weight (g)].

### Histopathological Analysis

All patients had primary tumor resection and lymph node dissection. Besides primary tumor and lymph nodes pathology, largest tumor diameter, venous invasion, angiolymphatic invasion, perineural invasion, surgical margins, and K-ras mutation status were evaluated in the pathology department.

### Statistical Analysis

The normality of the data distribution was assessed with the Shapiro-Wilk test. Non-parametric data were presented as median and minimum-maximum ranges, while parametric data as means ± standard deviation (SD). Nominal and categorical variables were presented as frequencies and percentages. The parametric distribution was compared with the Student t-test in independent groups, and with the Mann-Whitney U test in the rest. Survival was evaluated by the Kaplan-Meier method. Categorical variables were evaluated with chi-square and Fisher’s Exact Contingency tests. The tests were two-sided. p<0.05 was accepted as significant. Statistical analysis was performed with SPSS statistical software, version 21.0 (IBM SPSS Statistics for Windows, Version 21.0. Armonk, NY: IBM Corp.). The receiver operating characteristics (ROC) was applied to determine the threshold value for the optimal inventory SUV_max_. Sensitivity, specificity, positive-negative predictive values and accuracy rates were calculated for different threshold values.

## Results

**Figure 1:**
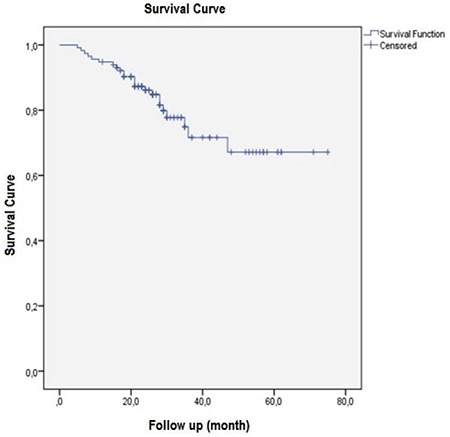
Kaplan-Meier survival analysis of all patients

We reviewed data of 115 patients that underwent ^18^F-FDG PET/CT for initial staging. Thirty nine (34%) patients were female and 76 (66%) patients were male. The mean ± SD of patients was 58.7±11.4 (range=31-82 y). All patients underwent surgery and their histopathologic diagnosis was rectal adenocarcinoma. According to TNM, 66 patients had pN stage 0, 37 had pN stage 1 and 20 had pN stage 2 disease. Eleven patients had stage 1, 42 had stage 2, 39 had stage 3 disease and 23 had metastatic disease in initial staging (Table 1) (8). Subsequent to initial ^18^F-FDG PET/CT scan, all patients were followed-up for a 12-75 (29.7) months period and the estimated 5-year survival was found to be 61.8 ±2.9 months (Figure 1). Increased pathological ^18^F-FDG uptake in primary tumor was observed on PET/CT in all patients. The mean ± SD SUV_max_ of the primary tumor was 21.0±9.1 (7.6-55). There was no significant relation between primary tumor SUV_max_ and disease-free survival (DFS) or primary tumor SUV_max_ and overall survival (OS) (p=0.760 and p=0.525) (Table 2).

Fifty eight out of 115 patients (50.4%) had locoregional lymph node with increased ^18^F-FDG uptake in initial PET/CT. Histologically proven metastatic LNs were found in 39 out of 115 patients (33.9%). Thirty of the PET/CT findings were true positive, 28 false positive, 48 true negative and 9 false negative. The sensitivity, specificity, positive predictive value, negative predictive value and accuracy of ^18^F-FDG PET/CT were 76.9%, 63.1%, 51.7%, 84.2% and 67.8%, respectively.

**Figure 2:**
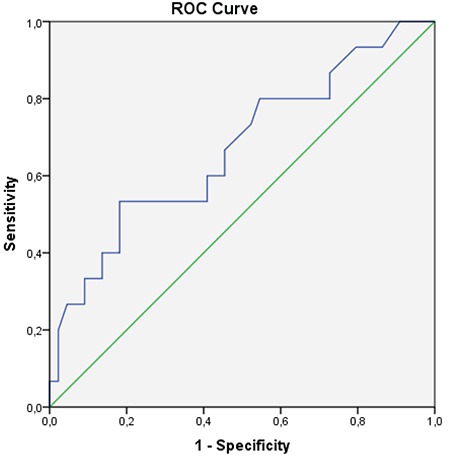
ROC analysis of lymph node SUV_max_ (blue) with DFS ROC: Receiver operating characteristic, SUV_max_: Maximum standard uptake value, DFS: Disease-free survival

The mean ± SD SUV_max_ of metastatic lymph nodes were 4.6±2.8 (1.7-14.9). There was a significant correlation between locoregional metastatic lymph node SUV_max_ and DFS or OS (p=0.049 and p=0.045, respectively). Also in accordance with the ROC curve (Figure 2) when the cut-off point for the lymph node SUV_max_ was taken as 3.55; the sensitivity and specificity were 66.7% and 54.5% for DFS, 72.7% and 54.2% for OS, respectively. Metastatic lymph node SUV_max_ was higher in the patient group who died compared with the individuals who survived [5.8 (2.5-14.9) vs 3.5 (1.7-12.0)].

The lymph node SUV_max_ was higher in the patients as the stage of the disease increased and it was found to be statistically significant (p=0.002). The mean ± SD SUV_max_ of the metastatic lymph nodes was 2.5±0.6 for stage 1, 3.2±1.8 for stage 2, 4.7±2.0 for stage 3 and 6.7±3.9 for stage 4.

K-ras mutation was assessed in 22 patients and 11 of patients had K-ras mutation (50%). There was no association between K-ras mutation and the primary tumor SUV_max_ or metastatic tumor SUV_max_ (p=0.358 and p=0.643, respectively). There was no correlation between tumor localization, angiolymphatic invasion, perineural invasion of the primary tumor and DFS or OS (p>0.05). A higher maximum diameter of the primary tumor and the number of metastatic lymph node or positive venous invasion of tumor predicted worse DFS in patients (p=0.012, p=0.03, p=0.024, respectively).

N-stage was determined to be significant on DFS and OS (p=0.001, p=0.021, respectively). According to TNM stage, 23 patients had metastases (17 liver, 10 thorax, 11 non-regional lymph nodes, 3 peritoneum and 1 bone metastases) at diagnosis. Metastatic disease had predictive value on both DFS and OS (p<0.001).

## Discussion

Rectal cancer is a common malignancy and a common cause of mortality; thus, accurate staging of rectal cancer is extremely important in determining the prognosis of the disease and the treatment protocol (9). The overall predicted 5-year survival rate after diagnosis is less than 60% but it is significantly dependent on disease stage (10).

One of the most used techniques to detect prognosis in clinical practice is ^18^F-FDG PET/CT (11); providing a semi-quantitative measure of the metabolism of the tumoral lesion that is a comparative rate of tumoral proliferation (12). In patients with lung cancer and esophageal cancer, the SUV_max_ is a predictive value for survival. Primary tumor ^18^F-FDG uptake in non-small cell lung cancer was demonstrated to be the most potent prognostic factor in the curative treatment patient group (13). As the same, the initial ^18^F-FDG uptake was predictive of survival in esophageal cancer (14). In the light of this data, primary tumor SUV_max_ of rectal cancer for predicting survival has been investigated in previous studies and SUV_max_ has been asserted as a prognostic factor (15). However, in a study to assess the prognostic value of preoperative ^18^F-FDG PET/CT, Lee et al. (16) detected that SUV_max_ was not a significant predictor of recurrence or DFS. In a rectal cancer patient group that received neoadjuvant radiation therapy, Oku et al. (17) found that pretreatment ^18^F-FDG PET/CT SUV_mean_ was unrelated to disease recurrence or response to therapy. In the study by Bang et al. (18), SUV_mean_, SUV_peak_, and SUV_max_ were not associated with recurrence or neoadjuvant radiation and chemotherapy in locally advanced rectal cancer. In a study of 100 patients by Deantonio et al. (7); higher level of metabolic parameters was significantly associated with higher clinical tumor stage that was considered as more aggressive, but none of the analyzed metabolic parameters had any significant correlation with DFS or OS. In a study conducted by Ogawa et al. (19); it was shown that OS did not differ significantly between low and high SUV_max_ and SUV_mean_ groups. On the other hand, Dehdashti et al. (20) found that high ^18^F-FDG uptake (≥14.3) on initial PET/CT correlated with better DFS and better neoadjuvant therapy response, and so on better outcomes contrary to prior studies. Regarding the patient results in our study, increased FDG uptake of the primary tumor was shown in all patients. As the preoperative ^18^F-FDG PET/CT parameters could be able to predict survival outcomes in rectal cancer, we evaluated the impact of primary tumor SUV_max_ on survival and it was shown that there was no correlation between primary tumor SUV_max_ and survival.

^18^F-FDG PET/CT is not widely used in routine practice, but is a useful test for the detection of metastatic lymph nodes and distant metastasis (21). As is well known, one of the most important prognostic factors in rectal cancer is lymph node metastasis (22) and correct diagnosis of lymph node metastasis in staging might improve the therapy (23). Bae et al. (24) evaluated the diagnostic accuracy of ^18^F-FDG PET/CT in diagnosis of lymph nodes in patients with rectal cancer with optimal SUV_max_ cut-off values according to lymph node size. It was shown that lower cut-off SUV_max_ improved the diagnostic performance of ^18^F-FDG PET/CT, especially in small lymph node evaluation. In our study, lymph node size was not considered in determining optimal SUV_max_ cut-off values but the lymph node metastasis revealed by ^18^F-FDG PET/CT was always confirmed histologically and the optimal cut-off values of the SUV_max_ were calculated using ROC analysis. As we took a cut-off SUV_max_ lymph node value of 2.5 in the ROC analysis regarding DFS (area under the curve 0.671; p=0.049; 95% CI: 0.506-0.836; Figure 2), the sensitivity and specificity at this value were 80.0% and 45.5%, respectively. Chen et al. (25) identified a cut-off SUV value of 1.15 in the ROC analysis regarding DFS and the sensitivity and specificity at that value were 84.2% and 59.2%, respectively, pointing out similar sensitivity but slightly higher specificity than the results of our study.

The other significant finding in our study was that the high SUV_max_ of the lymph nodes was predictive of low survival. In this respect, prior literature studies have put forward several and conflicting data about the relationship between survival and PET parameters such as primary tumor SUV_max_, MTV and post-treatment metabolic changes (7,18,26,27) Chen et al. (25) showed that preoperative SUV lymph node measured on ^18^F-FDG PET/CT could predict the recurrence in patients with colorectal carcinoma. In our study, we assessed only the patients with rectal carcinoma. Furthermore, in addition to DFS, OS was evaluated, as well. The higher level of lymph node SUV_max_ in preoperative ^18^F-FGD PET/CT was significantly related to the more advanced clinical stage of the disease that was associated with more aggressive disease and poor survival outcomes. Morphological criteria such as lymph node size and number, which were higher in advanced stages, were considerable in this issue due to partial volume effect in small lymph nodes <10 mm in size leading to underestimation of true SUV (28).

Bang et al. (18) showed that lymphatic and venous involvements were not significantly associated with 3-year DFS, whereas in our study positive venous invasion had a prognostic impact (worse DFS). Chen et al. (29) reported that the mutated K-ras tumors were associated with higher ^18^F-FDG accumulation and higher SUV_max_ was a predictor of K-ras mutations. We should note that in our study, due to the retrospective nature, only 20 patients’ K-ras results were interpreted and there was no correlation between K-ras results and ^18^F-FDG PET/CT metabolic parameters. Furthermore, it has been reported that there is a heterogeneity of K-ras status among the primary rectal tumor (30). As a result, the correlation analysis might be biased because of the small patient group and dissected specimens for mutational testing could not reflect the real status of the total tumor, and ^18^F-FDG PET/CT displayed the entire status of tumor (31).

In this retrospective study, we found that primary tumor SUV_max_ in preoperative staging ^18^F-FDG PET/CT scan had no effect on predicting survival. On the other hand, the SUV_max_ of lymph nodes in ^18^F-FDG PET/CT was predictive regarding survival. Our results showed that the staging system which was still used in clinical practice was effective on predicting survival of patients with rectal cancer.

### Study Limitations

There were some limitations in this study. First of all, it was a retrospective, single-institution study; thus, the findings of ^18^F-FDG-PET/CT as a prognostic factor in preoperative rectal cancer required strengthening in a prospective, multicenter study with a larger patient number. Another weakness was the small sample size of patients and the heterogeneity of the clinical stages of the disease in the patient group that might have biased the outcome of this study. Additionally, we chose the SUV_max_ cut-off values from the ROC curves for the balance of sensitivity and specificity. However, these choices could be improved with a larger and homogeneous patient group.

## Conclusion

The results showed that primary tumor SUV_max_ obtained in initial ^18^F-FDG PET/CT was not predictive of survival in patients with rectal cancer. On the other hand, the lymph node SUV_max_ had negative effect on survival. For this reason, we suggest the use of high lymph node SUV_max_ as a new parameter for clinical practice, which has a negative impact on survival.

## Figures and Tables

**Table 1:**
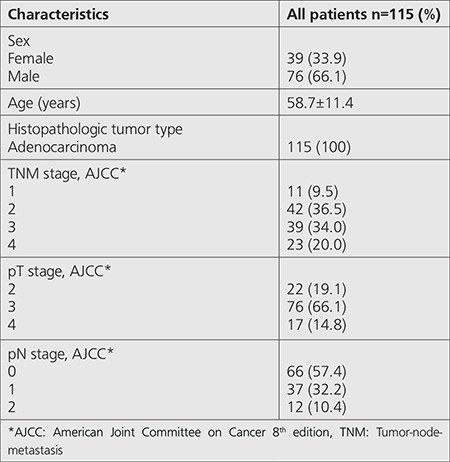
Patient characteristics

**Table 2:**
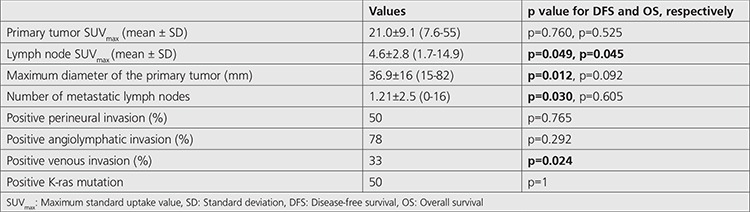
Correlation between histopathological features, SUV_max_ values and survival rates
